# Laser Interstitial Thermal Therapy for Posterior Fossa Lesions: A Systematic Review and Analysis of Multi-Institutional Outcomes

**DOI:** 10.3390/cancers14020456

**Published:** 2022-01-17

**Authors:** Mohammadmahdi Sabahi, Stephen J. Bordes, Edinson Najera, Alireza M. Mohammadi, Gene H. Barnett, Badih Adada, Hamid Borghei-Razavi

**Affiliations:** 1 Neurosurgery Research Group (NRG), Student Research Committee, Hamadan University of Medical Sciences, Hamadan 65141, Iran; m.sabahi@edu.umsha.ac.ir; 2 Department of Surgery, Louisiana State University Health Sciences Center, School of Medicine, New Orleans, LA 70112, USA; sbor10@lsuhsc.edu; 3 Department of Neurological Surgery, Pauline Braathen Neurological Center, Cleveland Clinic Florida, Weston, FL 33331, USA; najerae@amc.edu (E.N.); adadab@ccf.org (B.A.); 4 The Rose Ella Burkhardt Brain Tumor and Neuro-Oncology Center, Cleveland Clinic, Cleveland, OH 44195, USA; MOHAMMA3@ccf.org (A.M.M.); BARNETG@ccf.org (G.H.B.); 5 Department of Neurosurgery, Neurological Institute, Cleveland Clinic, Cleveland, OH 44195, USA

**Keywords:** laser interstitial thermal therapy, LITT, neuro-oncology, posterior fossa, systematic review

## Abstract

**Simple Summary:**

Laser interstitial thermal therapy (LITT), a minimally invasive stereotactic treatment option, is emerging as a viable treatment option for deep-seated primary and metastatic brain lesions due to the use of real-time magnetic resonance thermography. LITT has been used with good outcomes for a variety of brain lesions. However, LITT for posterior fossa lesions remains understudied. Currently, there are no treatment recommendations for the LITT of the posterior fossa due to a limited pool of data. In this study, we analyze multi-institutional outcomes of LITT for the treatment of posterior fossa lesions based on the demographics of the samples, including age and gender distribution, tumor pathology, presence or absence of prior treatment, tumor volume assessment, and complications; discuss current results; and suggest areas of future study.

**Abstract:**

Background: Laser interstitial thermal therapy (LITT) has emerged as a treatment option for deep-seated primary and metastatic brain lesions; however, hardly any data exist regarding LITT for lesions of the posterior fossa. Methods: A quantitative systematic review was performed. Article selection was performed by searching MEDLINE (using PubMed), Scopus, and Cochrane electronic bibliographic databases. Inclusion criteria were studies assessing LITT on posterior fossa tumors. Results: 16 studies comprising 150 patients (76.1% female) with a mean age of 56.47 years between 2014 and 2021 were systematically reviewed for treatment outcomes and efficacy. Morbidity and mortality data could be extracted for 131 of the 150 patients. Death attributed to treatment failure, disease progression, recurrence, or postoperative complications occurred in 6.87% (9/131) of the pooled sample. Procedure-related complications, usually including new neurologic deficits, occurred in approximately 14.5% (19/131) of the pooled sample. Neurologic deficits improved with time in most cases, and 78.6% (103/131) of the pooled sample experienced no complications and progression-free survival at the time of last follow-up. Conclusions: LITT for lesions of the posterior fossa continues to show promising data. Future clinical cohort studies are required to further direct treatment recommendations.

## 1. Introduction

Neurosurgeons and neuro-oncologists have considerable difficulties when it comes to surgical treatment of deep or difficult-to-access lesions in both pediatrics and adults. Approaching deep subcortical disorder has a high morbidity rate, which is a critical issue in different types of surgeries, including epilepsy surgery, vascular surgery, and tumor surgery [1].

Laser interstitial thermal therapy (LITT), a minimally invasive stereotactic treatment option, first introduced in 1983, is emerging as a viable treatment option for deep-seated primary and metastatic brain lesions due to the use of real-time magnetic resonance (MR) thermography [2]. The therapy involves the insertion of a laser catheter, which is subsequently heated to destroy pathologic tissue [3]. Real-time MR guidance focuses the laser treatment to decrease damage to surrounding structures [4]. LITT has been used for epilepsy treatment, which is not treatable with stereotactic radiosurgery (SRS), and also radiation necrosis (RN), which does not respond to SRS or surgery [5].

In addition, LITT is of particular interest to surgical neuro-oncologists since there is evidence supporting additional LITT effects that can be used to augment adjuvant therapies, such as disruption of the blood–brain barrier (BBB), which facilitates the dissemination of chemotherapeutics [6,7].

However, LITT for posterior fossa lesions remains understudied. The posterior fossa is a small cranial compartment, and anatomical challenges require significant surgical consideration [8]. Currently, there are no treatment recommendations for LITT of the posterior fossa due to a limited pool of data. In this study, we analyze multi-institutional outcomes of LITT for the treatment of posterior fossa lesions, discuss current results, and suggest areas of future study.

## 2. Materials and Methods

The following study question was used to perform this systematic review, which followed the Preferred Reporting Items for Systematic Reviews and Meta-Analyses (PRISMA) guidelines: Is laser interstitial thermal therapy (LITT) safe and effective in the treatment of tumors in the posterior fossa? [9,10]. Since we tried to get the PRISMA registration code after the data extraction, our project was not eligible for a registration code based on the International Prospective Register of Systematic Reviews’ (PROSPERO) new guideline.

### 2.1. Literature Search and Selection Criteria

We reviewed published articles in English with no limits on the year of publication. To identify information on the efficacy and safety of LITT in posterior fossa tumors, the following databases were searched: MEDLINE (through PubMed), Scopus, and Cochrane. The following are some of the keywords and terms that were used in this study: “Laser Interstitial Thermal Therapy” OR “LITT” OR “Laser ablation” AND “brainstem” OR “cerebellum” OR “posterior fossa” OR “posterior cranial fossa” OR “medulla oblongata” OR “pons” OR “midbrain”. The most recent search took place in September 2021. Furthermore, all relevant cited references in the original publications were reviewed to identify articles that were not indexed by the databases listed. EndNote X7.1 (Thomson Reuters, Toronto, ON, Canada) was used to aggregate and screen the papers.

The following inclusion criteria were used to make the final selection: (1) case series and prospective and retrospective studies evaluating LITT on posterior fossa tumors and (2) studies that presented LITT outcomes in posterior fossa tumors. (1) Animal studies, letters to the editor, expert opinions, and commentaries and (2) studies that only delineated LITT on tumors other than the posterior cranial fossa were all excluded.

Two independent reviewers (M.S. and S.B.) evaluated the relevance of 104 publications found by this query. In the initial search, 33 articles in MEDLINE (PubMed), 79 records in Scopus, and 2 records in Cochrane were found. Following the removal of 31 duplicate articles, titles, and abstracts, 73 records were reviewed, 43 of which were irrelevant. This resulted in a final list of 30 articles that were assessed for eligibility. From the total of 30 records, 6 papers were eliminated: 4 review articles and 2 letters to the editor.

**Figure 1:**
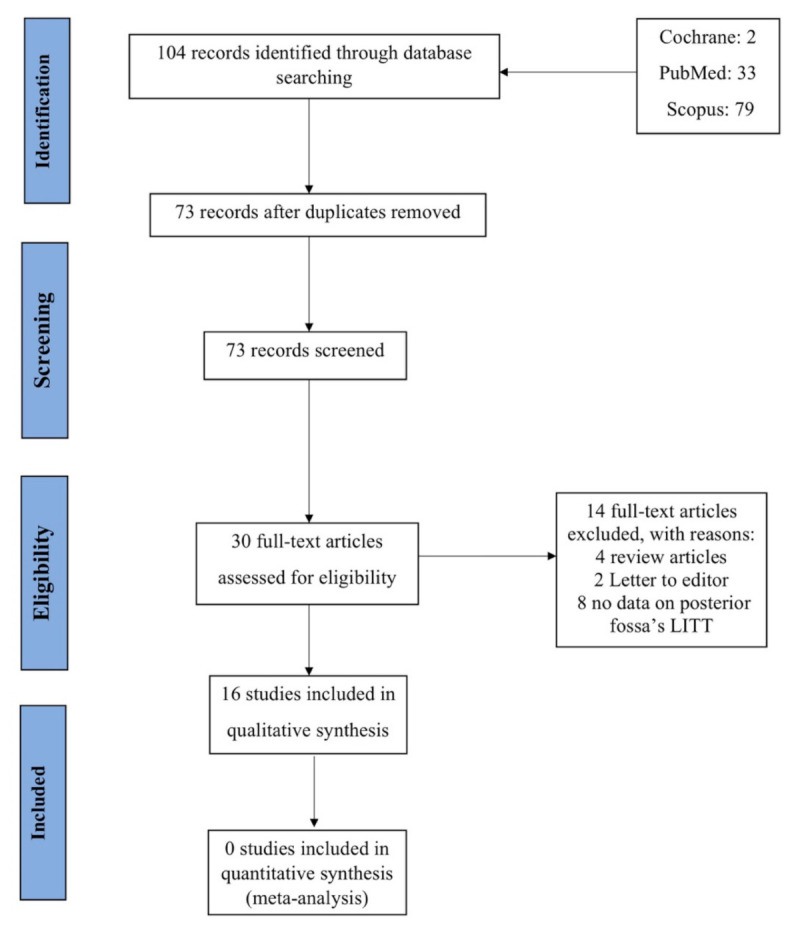
Preferred Reporting Items for Systematic Reviews and Meta-Analyses (PRISMA) diagram of included studies.

Eight papers were excluded from the remaining 24 since they lacked data on posterior fossa tumors that underwent LITT. As a result, 16 studies were included in this study; and the search strategy is summarized in Figure 1.

### 2.2. Data Extraction

Data extraction was performed based on a predefined protocol by one author (M.S.) and was rechecked by another one (S.B.). Disagreements were resolved by a third author (E.N.). The extracted data included (1) demographics of the samples, including age and gender distribution of the patients, male (M) and female (F), who underwent LITT, (2) data related to the tumor pathology, (3) presence or absence of prior treatment, including SRS, resection through craniotomy, radiotherapy, or a combination of the aforementioned therapies, such as radiotherapy + SRS, resection + SRS, resection + SRS + radiotherapy, or radiotherapy + resection; (4) tumor volume assessment, including pre-LITT, post-LITT, and at follow-up; (5) complications, and (6) Karnofsky performance status (KPS) score both pre-LITT and post-LITT. Any other data not relevant to the aim of this systematic review were ignored.

Various techniques, such as OsiriX MD (Pixmeo SARL, Geneva, Switzerland) [11,12] and iplan workstation (BrainLab, Munich, Germany) [13,14,15], were used in different studies to provide tumor dimensions and volumetric analyses.

Since LITT in posterior fossa tumors is a rather uncommon entity, with the majority of data coming from small sample sizes and a lack of high-power studies, we included case reports and case series in the analysis.

## 3. Results

Data selection was also in concordance with PRISMA guidelines [10]. We designed a table for the extraction of the relevant data from the included studies (Table 1). Data extraction was performed by one author (M.S.) and rechecked by another one (S.B.). In case of any disagreement, a third person intervened to finalize the decision (E.N.).

A total of 150 patients with a mean age of 56.47 years had undergone LITT for posterior fossa tumors between 2014 and 2021 (Table 1) [11,13,14,15,16,17,18,19,20,21,23,24,25,26]. Of these patients, 76.1% were female and 23.9% were male. Data reported for 104 of these patients showed a combined mean pre-LITT lesion volume of 3.93 cm^3^, while in the subgroup that had long-term follow-up, the mean pre-LITT lesion volume was 2.92 cm^3^. The post-LITT lesion volumes were reported for 79 of the patients, with a combined mean volume of 6.16 cm^3^. Of these, 40 patients had an additional post-LITT volume measured at their last follow-up 3 months after their LITT. The combined mean volume for this subgroup of patients was 2.58 cm^3^. Data for posterior fossa residual volume were not widely or reliably reported among studies for follow-up imaging after 3 months. Two studies did not report tumor volume, and they only reported the length and width of the tumor (cm^2^), and since it was not possible to combine these data with the previous ones (cm^3^), the tumor dimensions in these articles were not included in our mentioned calculations [19,21]. One study reported only the diameters of the lesions on posterior fossa and was also excluded in pre-LITT volume’s measurement [17]. Three studies reported pre- and postprocedural KPS scores, with a combined mean score of 90 pre-LITT and 80 post-LITT for 38 patients [12,14,15].

For 111 posterior fossa lesions, primary pathology was reported and included RN (*n* = 7); breast cancer (*n* = 34); brain lesions; including both tumoral and vascular abnormalities and also epileptic focus (*n* = 28); lung cancer (*n* = 22); renal and gastrointestinal cancer (*n* = 7); and metastasis from other sources (*n* = 13). For 134 of the patients, prior treatment regimens were stated and included prior resection (*n* = 10), SRS (*n* = 82), radiotherapy (*n* = 2), combination therapy (*n* = 25), and no prior treatment (*n* = 15).

For 128 posterior fossa lesions, the location was reported and included the cerebellum (*n* = 110), the vermis (*n* = 1), the cerebellar peduncle (*n* = 3), the brain stem (*n* = 13), and the pineal region (*n* = 1)

Morbidity and mortality data could be extracted for 131 of the 150 patients included in this study. Nine deaths (9/131) resulted from disease progression, treatment failure, or metastasis, and no death occurred from LITT treatment [11,25]; 103 patients had no procedural complications and showed stabilization of disease at the time of last follow-up, which ranged from 2 to 24 months [11,14,15,16,17,18,19,20,23,25]; and 19 patients developed intra- or postoperative complications, including transient and permanent neurologic deficits [11,12,14,21,23,25]. Procedure-related complications, usually including new neurologic deficits, occurred in approximately 14.5% (19/131) of the pooled sample. Neurologic deficits improved in most cases either in combination with steroid treatment and rehabilitation or spontaneously, typically within 6 months, and included either complete or partial relief of motor, sensory, and visual disturbances; alleviation of unsteady gait and ataxia; and enhancement in speech-related disorders, such as aphasia, slurred speech, and scanning speech. Assessment of the data demonstrates that the main determining factor in the occurrence of complications after the LITT is the proximity of the lesion to the cranial nerves. Furthermore, it seems individuals with brainstem lesions experienced higher complications, whereas ablation of the cerebellum resulted with a lower risk of complications. In comparison with patients without complications, those who had postoperative complications had smaller postablation-to-preablation tumor volume ratio.

Of the pooled sample, 78.6% (103/131) experienced no complications and progression-free survival at the time of last follow-up, which ranged from 2 to 24 months depending upon the individual study.

## 4. Discussion

We systematically reviewed all available studies regarding LITT for posterior fossa lesions, extracted all reported data, pooled the data to increase study power, and reported available morbidity and mortality rates.

When contemplating LITT for this patient population, we propose a thorough evaluation of the maximum attainable ablation by analyzing feasible trajectories and proximity to key neuroanatomical structures, related perioperative risks, and potential benefits.

Eleven articles specifically examine the use of LITT on posterior fossa lesions. Among them, Tan et al. [18], Chan et al. [19], Eliyas et al. [20], Kozlowski et al. [22], and Lawrence et al. [21] each reported only 1 patient who underwent LITT for their posterior fossa lesions, which primarily originated from unresectable intrahepatic cholangiocarcinoma [18], anaplastic astrocytoma [19], pulmonary adenocarcinoma with recurrent metastasis [20], poorly differentiated carcinoma consistent with gastrointestinal versus pulmonary metastasis [22], and hemorrhagic pontine cavernoma [21], respectively. With the exception of the last case, which developed diplopia secondary to Abducens’ palsy [21], none of the aforementioned studies developed post-LITT complications [18,19,20]. Gamboa et al. reported MR-thermography-guided LITT for 2 brainstem cavernous malformations [17]. Both patients demonstrated remarkable symptomatic improvement and were hemorrhage-free at 12- and 6-month follow-up, respectively. In our systematic review, there are 4 case series that exclusively discuss the use of LITT on posterior fossa tumors: in 58 patients with 60 lesions [11], in 13 patients [15], in 8 patients [14], and in 4 patients [16]. In addition to the items mentioned in Table 1, Ashraf et al. reported that an 84% overall local control rate was achieved at 9.5-month median follow-up [11]. No mortality was associated with the procedure in three studies [14,15,16], while there was 1 procedure-related death in the Ashraf et al. study [11]. The median volume of the ablation cavity and perilesional edema gradually decreased in follow-ups [14,15]. Luther et al. stated that radical ablations are both possible and safe in the posterior fossa. Immediately after surgery and at the time of the final follow-up, radical ablations may result in higher reductions in perilesional edema and an enhanced functional status [12].

Five papers used LITT for lesions in multiple brain locations, including some cases in the posterior fossa [13,23,24,25,26]. Dadey et al. presented a 55-year-old male and a 36-year-old female both with gangliogliomas whose lesions were located on the right dorsal pons and the left medial cerebellum, respectively [23]. They underwent LITT in which STarFix platforms (FHC Worldwide), a customized stereotactic platform, was used for probe insertion. Among all 36 patients with 50 lesions in Beechar et al. who underwent LITT, 4 lesions were in the cerebellum [13]. Among the 16 patients who experienced neurological complications, 2 patients had cerebellar lesions. Additionally, Beechar et al. did not divide the mean age, gender distribution, and KPS score of posterior fossa tumors from other tumors. The overall demographic information in this study was as follows: 51 years (mean age), 16M/20F (gender), and 80 (KPS score) in both pre-LITT and post-LITT, respectively [13]. Median T1-weighted MRI with contrast volume over time in all patients showed a gradual decrease in tumor volume post-LITT over a 3–12-month period [13]. Ahluwalia et al. reported 6 patients with cerebellar lesions who underwent LITT while their other 36 patients had lesions in areas other than the posterior fossa [24]. Similar to the Beechar et al. [13] study, Ahluwalia et al. [24] did not specify the mean age, the gender distribution, or the KPS score in posterior fossa lesions. The overall demographics for all lesions were 58.5 years (mean age), 15M/27F (gender), and 85 (KPS score) in both pre- and post-LITT, respectively. Primary tumor pathologies in this study included NSCLC in 43% of patients, breast cancer in 17%, melanoma in 10%, and other tumor forms in 30%. Based on the results of the data analysis, successful LITT can result in the KPS score stabilizing. Moreover, as previously proven, individuals with both brain metastases and presumptive posterior fossa RN respond effectively to ablation treatment, suggesting that LITT may be a viable therapeutic approach for both brain metastasis and RN [27]. This is particularly important since brain metastasis and RN’s imaging demonstrations are ambiguous and require tissue biopsy to confirm the diagnosis. Shao et al. reported 238 patients with different brain tumors who underwent LITT in a single institution [26]. Among them, 9 patients had posterior fossa lesions, of which 1 patient was treated prior to 2015 (cohort 1) and the remaining 8 patients were treated between 2015 and 2018 (cohort 2). In this study, LITT indications comprised gliomas (70.2%), RN (21.0%), and metastasis (8.8%). Overall, a comparison of patient demographics between 2 cohorts revealed that with the exception of age, which was higher in cohort 2, operative time, permanent motor deficits, and 30-day mortality were significantly lower in cohort 2, while other data points remained consistent over time. A low KPS score correlated with incremented permanent deficits [26]. In terms of LITT complication, thermal-ablation-related complication (hyperthermia, post-ablation edema, or ablation-induced bleeding) and laser-insertion-related complication are the two types of postoperative complications associated with LITT [28,29,30]. In this systematic review, death attributed to treatment failure, disease progression, recurrence, or postoperative complications occurred in approximately 6.8% (9/131) of the pooled sample. Procedure-related complications, including new neurologic deficits, occurred in approximately 14.5% (19/131) of the pooled sample.

In comparison to other therapeutic modalities in the posterior fossa, LITT seems to be safe and less complicated. Surgical complications were seen in 38.5% of patients with giant solid hemangioblastomas in the posterior fossa according to the Jeon et al. study [31]. Siomin et al. reported leptomeningeal disease after surgical removal of posterior fossa metastases in 50% of patients, whereas leptomeningeal disease occurred after SRS in 6.5% of patients [32]. However, no case of leptomeningeal disease has been observed after LITT for posterior fossa metastasis.

A study by Kaye et al. used LITT for in-field recurrence of brain metastasis after SRS. In this study, 70 patients with 97 lesions underwent LITT [25]. Among these lesions, 20 lesions were cerebellar and 2 lesions were pontine. Similar to two studies described previously [13,24], demographic data for posterior fossa tumors were not expressed separately. According to the overall treatment description, the mean age and the gender distribution were 62.5 years and 20M/50F, respectively [25]. In terms of outcomes, the study separated the patients who died into 2 major groups: (A) patients with neurologic death (20 patients) and (B) patients with non-neurologic death attributed to other causes (24 patients). In group A, there were 6 cerebellar lesions and 2 brainstem lesions, while group B had 9 cerebellar lesions [25]. The overall KPS scores in group A and group B were 70 and 79, respectively [25]. However, low KPS is a known risk factor for neurologic death among patients with brain metastasis [33]. Based on etiology, the authors further subdivided group A (patients with neurologic death) into 4 sub-groups: (C) local recurrence (7 patients with 10 LITT procedures), (D) distant intracranial progression (5 patients with 10 LITT procedures), (E) recovery failure (7 patients with 9 LITT procedures), and (F) postoperative complications (1 patient) [25]. Cerebellar lesions accounted for 4/10 and 2/10 lesions in groups C and D, while pontine lesions accounted for 1/9 and 1/10 in groups E and D [25]. NSCLC was the prevalent tumor pathology in groups C and E, while breast cancer was the prevalent pathology in group D. The higher rate of neurologic mortality in patients who had lesions on the brainstem was not an unexpected finding since the risk of any intervention in these deep and eloquent locations remains high. The difference in the rate of tumor volume reduction after LITT depends more on the underlying histopathology of the tumor than on the size and location of the tumor [11].

Traylor et al. discovered a remarkable correlation between patients who received adjuvant chemotherapy (targeted and systemic) after LITT and a prolonged time to local development, suggesting that systemically administrated chemotherapeutic drugs have restricted penetration of the BBB [15]. This is due to LITT’s hyperthermic disruption of the BBB, which is comparable to a mechanism reported previously that may improve chemotherapeutic drug delivery to the central nervous system (CNS) [6]. These data imply that combining chemotherapy with LITT for the treatment of posterior fossa lesions might be a potential therapeutic approach.

According to our review, there were 6 records (6/131) of posterior fossa syndrome (PFS), including 2 cases of slurred speech [11], 1 case of scanning speech [11], 1 case of speech impairment [12], 1 case of dysarthria [23], and 1 case of dysarthria due to a new lesion [16]. Khan et al. reported that 34% of their medulloblastoma patients who received surgical resection had PFS. They further assessed their patients once they finished craniospinal radiotherapy and every 3–6 months after that [34]. At 1 year, 14.8% and 7.7% of children with PFS1 and PFS2 had severe dysarthria, respectively [34]. Gentile et al. reported that 23.1% of patients with posterior fossa tumor who underwent proton beam therapy demonstrated PFS [35]. In comparison with these two mentioned studies, LITT has less probability (4.5%) of evoking PFS and thus seems to be a safer therapeutic modality.

This systematic review highlights a number of the difficulties that are faced for LITT of the posterior fossa due to its delicate anatomical area. Because of the close proximity to the dural venous sinuses, the brain stem, and the fourth ventricle, meticulous trajectory planning is required to ensure appropriate ablation coverage. As a result, in some studies, certain lesions were not susceptible to full ablation and caused metastasis or recurred.

Overall, reported morbidity, mortality, and disease progression rates were low for LITT of the posterior fossa.

### Limitations and Future Research

Small sample sizes in most studies, heterogeneous tumor types, thinness of MRI slices used to evaluate volume measurements in some studies, patients lost to follow-up, lack of high-power studies, and insufficient power to adequately prove the efficacy or safety profile of LITT for any single diagnosis are all limitations of this systematic review. 

Further randomized controlled studies with larger patient sample sizes and adequate follow-up are needed to further validate the efficacy of LITT for posterior fossa lesions with regard to patient pathology and prior treatment.

## 5. Conclusions

The use of LITT for the treatment of posterior fossa lesions continues to show promise. LITT is a feasible method for the treatment of deep-seated lesions of the posterior fossa, and pooled data from this study show that morbidity and mortality rates are relatively low. Data included in this study warrant future clinical cohort studies to draw definitive conclusions and direct treatment recommendations regarding the use of LITT for lesions of the posterior fossa.

## Figures and Tables

**Table 1:** LITT on posterior fossa tumors.

Author, Year, (Ref.)	Sample Size	Mean Age	Histology	Location	Prior Treatment	Volume (Mean ± SD)			Complications	KPS	
						Pre-LITT Tumor Volume	Post-LITT Cavity Volume	Tumor Volume at Last Follow-Up		Pre-LITT	Post-LITT
Traylor, 2019, [15]	13	58	RN: 5, breast: 4, lung ^†^: 2, kidney and GI ^‡^: 2	NR	SRS: 8, RT: 1, Co ^$^:4	4.63 ± 2.85 cm^3^	6.90 ± 3.42 cm^3^	3.14 ± 1.39 cm^3^	CN7&8 palsy: 1	90	80
Borghei-Razavi, 2018, [14]	8	53.87	RN: 2, brain *: 3, lung ^†^: 1, others ^§^: 2	Cerebellum: 6 Cerebellar peduncle: 2	Res:1, SRS: 2, N: 5	5.58 ± 5.27 cm^3^	9.64 ± 7.33 cm^3^	5.67 ± 8.39 cm^3^	Wound infection: 1, ataxia and hydrocephalus: 1, and CN6 palsy: 1	90	80
Eichberg, 2017, [16]	4	54.25	Breast: 3, others ^§^: 1	Cerebellum: 4	SRS: 1, Co ^$^: 3	3.35 ± 2.72 cm^3^	NR	NR	Diplopia: 1,dysarthria due to a new lesion: 1	NR	NR
Ashraf, 2020, [11]	58 ^¶^	56.4	Breast: 19 ^#^, brain *: 16, lung ^†^: 17, kidney and GI ^‡^: 2, others ^§^: 6	Cerebellum: 52 Brain stem: 7 Pineal region: 1	Res: 6, SRS: 34, RT: 1, Co ^$^: 12, N: 4	2.24 ± 0.21 cm^3^	3.92 ± 0.28 cm^3^	NR	Hemiparesis: 1, CN7 palsy: 1, facial droop and hemiparesis: 2, arm weakness: 1, dysmetria and slurred speech: 2, diplopia: 1, refractory cerebral edema: 1, hearing loss: 2, truncal ataxia and scanning speech: 1, and death: 1	NR	NR
Luther, 2021, [12]	17	57.9	Breast: 8, brain *: 3, lung ^†^: 1, kidney and GI ^‡^: 2, others ^§^: 3	Cerebellum: 16 Vermis: 1	Res: 1, SRS: 10, Co ^$^: 5, N: 1	2.0 ± 1.5 cm^3^	4.8 ± 2.2 cm^3^	1.7 ± 0.9 cm^3^	Diplopia: 1 and speech impairment: 1	91.2	NS
Gamboa, 2020, [17]	2	57.5	Brain *: 2	Brain stem: 2	Res: 2	1.8 and 1.6 cm	NR	NR	Left-sided weakness and ataxia: 1	NR	NR
Tan, 2020, [18]	1	71	Kidney and GI ^‡^: 1	Cerebellum: 1	N: 1	0.7 cm^3^	NR	Resolve	NR	NR	NR
Chan, 2016, [19]	1	60	Brain *: 1	Cerebellar peduncle: 1	SRS: 1	2.4 × 2.7 (cm^2^)	NR	Resolve	NR	NR	NR
Eliyas, 2014, [20]	1	67	Lung ^†^: 1	Cerebellum: 1	N: 1	3.23 cm^3^	NR	NR	NR	NR	NR
Lawrence, 2021, [21]	1	20	Brain *: 1	Brain stem: 1	N: 1	2.4 × 2.6 (cm^2^)	NR	1.3 × 1.2 (cm^2^)	Diplopia secondary to CN6 palsy: 1	NR	NR
Kozlowski, 2021, [22]	1	75	Others ^§^: 1	Cerebellum: 1	Co ^$^: 1	NR	NR	NR	NR	NR	NR
Dadey, 2016, [23]	2	45.5	Brain *: 2	Cerebellum: 1 Brain stem: 1	N: 2	12.85 ± 5.8 cm^3^	NR	NR	Internuclear ophthalmoplegia, right eye ophthalmoplegia, dysarthria, and reduced sensation on left side: 1	NR	NR
Beechar, 2018, [13]	4	NS	NS	Cerebellum: 4	SRS: 4	NS	NS	NS	Neurological complication: 2	NS	NS
Ahluwali, 2019, [24]	6	NS	NS	Cerebellum: 6	NS	NS	NS	NS	NS	NS	NS
Kaye, 2020, [25]	22	NS	NS	Cerebellum: 20 Brain stem: 2	SRS: 22	NS	NS	NS	Neurologic death: 8 Non-neurologic death: 10	NS	NS
Shao, 2020, [26]	9	NS	NS	NS	NS	NS	NS	NS	NS	NS	NS

* Brain consists of low-grade glioma (pilocytic astrocytoma, ganglioglioma, etc.), anaplastic astrocytoma, high-grade glioma (glioblastoma), ependymoma, hemangioblastoma, neuroendocrine tumor, pineal parenchymal tumor, epilepsy focus, and cavernous malformations. ^†^ Lung consists of small-cell lung carcinoma (SCLS), non-small-cell lung carcinoma (NSCLC), and pulmonary adenocarcinoma. ^‡^ Kidney and GI consists of renal cell carcinoma (RCC), colon cancer, and cholangiocarcinoma. ^§^ Others consists of ovarian cancer, cervical cancer, melanoma, parotid adenoid cystic carcinoma, unknown origin, and metastatic adenocarcinoma. ^$^ Co (combination) includes radiotherapy + SRS, resection + SRS, resection + SRS + radiotherapy, or radiotherapy + resection. ^¶^ There were 58 patients with 60 tumors. ^#^ Including one case of untreated metastatic breast cancer. Abbreviations: M: male, F: female, GI: gastrointestinal, CN: cranial nerve, RN: radiation necrosis, Res: surgical resection, RT: radiotherapy, Co: combination therapy, N: none, NR: not reported, NS: not specified.
